# Integrating knowledge, skills, and attitudes: professional training required for virtual reality therapists in palliative care

**DOI:** 10.3389/fmedt.2023.1268662

**Published:** 2023-10-02

**Authors:** Olive K. L. Woo

**Affiliations:** Department of Psychology, The University of Hong Kong, Hong Kong, Hong Kong SAR, China

**Keywords:** virtual reality, therapist, palliative care, adverse effect, professional training, knowledge, skill, attitude

## Abstract

Fully immersive virtual reality (VR) is an advanced technology increasingly studied and used in palliative care for symptom management. While the findings shed a positive light on its therapeutic potential, VR carries adverse effects, leading to ethical concerns. Based on the clinical experiences of a registered clinical psychologist who is also a certified thanatologist, we put forward a perspective on the importance of professional training for VR therapists in view of the possible risks posed by VR in palliative care. We propose professional trainings on knowledge, skills, and attitudes to ensure patients’ safety while maximizing the therapeutic benefits of VR. Given the scarcity of reports on such an area, we hope this perspective article opens up discussions and contributes to current understanding and emerging future directions to ensure quality and ethical delivery of VR in palliative care.

## 1. Introduction

Patients under palliative care often experience a variety of physical symptoms and psychological distress, which includes depression, anxiety, feelings of isolation, and a poor quality of life (1, 2). Despite the pharmacological and psychological interventions, patients encounter unmet needs such as unresolved pain, a low sense of control, and self-determination (3, 4). Thanks to advanced technology, virtual reality (VR) opens up a variety of therapeutic options with the potential to improve symptom burdens through immersion in a virtual environment (5–7). Three recent systematic reviews consistently report VR as acceptable and feasible, with potential therapeutic effects on symptom management in palliative care (8–10). Participants from various studies agreeably reported the feeling of “being away” from their current end-of-life conditions, which may help relieve their physical and psychological symptoms (11–13).

While such findings support the therapeutic use of VR, VR carries adverse effects, leading to ethical concerns. To minimize adverse effects, we put forward a perspective on the importance of professional training for VR therapists to ensure participants’ safety while maximizing the therapeutic benefits of VR. We propose professional trainings on knowledge, skills, and attitudes to ensure patients’ safety while maximizing the therapeutic benefits of VR. The proposed training hopes to address the imminent concern on the knowledge transfer that supports VR wide adoption in palliative care, which has been raised by Nwosu et al. (14). This perspective article is based on the literature support and the clinical experiences of the author, who is both a registered clinical psychologist and a certified thanatologist with more than 3 years of clinical experience using VR for therapeutic purposes in a palliative care setting. A novel VR psychological intervention named Flourishing-Life-Of-Wish Virtual Reality Therapy (FLOW-VRT®) has been developed, studied, and practiced (15, 16). It is a brief, manualized, and individualized psychological intervention specifically designed for patients under palliative care, theoretically based on flow theory (17), stress coping theory (18), self-determination theory (19), and attention restoration theory (20, 21). FLOW-VRT with a focus on relaxation (FLOW-VRT-Relaxation) allows patients to choose their preferred VR relaxation experience. Our case report (22) provided encouraging initial support for its feasibility, acceptability, and therapeutic potential as a cost-effective, scalable, and personalized VR relaxation. The preliminary result of our randomized controlled trial on 128 patients found that FLOW-VRT-Relaxation demonstrated significantly greater reductions in overall palliative symptoms when compared to traditional face-to-face relaxation (15).

## 2. Adverse effects of employing VR in palliative care

Adverse effects were defined as one's subjective experience of negative or harmful outcomes. Among the risks commonly documented in the literature, one prominent concern is “cybersickness,” (23–26), the symptoms of which include “nausea and vomiting, cold sweating, pallor, salivation, drowsiness, dizziness, headache, eye strain, lethargy, lack of initiative and chronic fatigue (23). Despite extensive research on its physical adverse effects, there remains a dearth of exploration and investigation into the psychological adverse effects posed by VR exposure, the significance of which has been highlighted by a recent systematic review conducted by Lundin et al. (27). In our clinical experiences, we observed that patients in palliative care are at higher risk of encountering difficulties during and after the transition into the real world after VR exposure, which were referred to as a reentry problem, as proposed by Behr et al. (28). It relates to the fact that patients in palliative care commonly opt for virtual experiences that align with their desired identities. For instance, a patient with a loss of walking ability chose to virtually hike on Lion Rock, a mountain in Hong Kong. On the one hand, such a temporary escape allows wish fulfillment in a virtual way; on the other hand, patients who exhibit a low level of acceptance towards their current palliative status may face additional challenges when re-entering their real-life patient role from their ideal virtual identity. This difficulty in transition may potentially lead to a heightened sense of hopelessness or helplessness, resulting in psychological distress.

## 3. Professional training required

To minimize the aforementioned risks while maximizing the therapeutic benefits of VR, we propose professional training for VR therapists. As a VR therapist takes on various roles, including clinical, technical, and supervisory roles, we will discuss further the required skills, knowledge, and attitudes for each role. The key components of competence for a VR therapist in terms of knowledge, skills, and attitudes are listed in Table 1.

**Table 1:** Professional training on skills, knowledge, and attitudes for VR therapists.

Roles	Skills	Knowledge	Attitudes
Clinical Role	• Deliver need and risk assessment • Deliver psychological interventions	• Knowledge on palliative care, counselling, psychology, or disciplines related to mental health • Knowledge on ethics	• Reasonable expectations towards VR efficacy • Empathy and respectfulness towards individual differences • Readiness to deliver timely psychological interventions • Ethical awareness • Continual Learning
Technical Role	• Know how to operate the hardware and software • Troubleshoot technical problems • Know how to minimize possible physical harms • Know how to select appropriate VR content for therapeutic purposes	• Basic knowledge on VR technology • Brief history and evolution of VR for therapeutic purpose • Update on development of VR technologies and evidence-based research under palliative care • Knowledge on the possible impact of VR technology • Knowledge on adverse effects • Knowledge on VR safety measures • Knowledge on data privacy and security	• Readiness and open-mindedness to equip knowledge and skills in field of VR technology • Adaptability in face of quick changes in technology, research and practices • Continual Learning
Supervisory Role	• Train and supervise VR therapists and VR assistants • Give appropriate feedback and guidance	• Technical knowledge on VR • Clinical knowledge on VR and psychological interventions	• Openness • Supportiveness • Respectfulness • Reflectiveness

### 3.1. Clinical role

The clinical role refers to the duties of providing psychological support along with VR interventions. As the field of psychology has been widely acknowledged for its crucial contribution to the development and advancement of high-quality VR for various purposes (29), we agree on the need for professional backgrounds in psychology, counseling, or other relevant mental health disciplines for the competency of VR therapists. First, we propose psychological assessments before VR to screen out patients with high risks of adverse effects. Prior psychological assessment refers to evaluations of the patient's (1) current mood state, as patients with depressive mood may appraise neutral events or stimulation in a negative and biased way and be prone to additional emotional disturbances; (2) current needs, i.e., physical, psychological, social, or spiritual; (3) degree of acceptance towards palliative status; and (4) psychological readiness for VR exposure to experience a favorable identity. Psychological assessment can be conducted through clinical interviews with patients to identify the individual's needs and evaluate the degree of illness acceptance. Assessment on mood state can be performed through clinical observations, collecting collateral information from ward staff or significant others, and data from a medical file as well. Second, relevant knowledge on psychology or related mental health disciplines may help select or design VR interventions that are specifically tailored to the unique needs and preferences of individual patients, upon assessment of one's psychological states, specific concerns and goals. Third, professional trainings on psychological interventions support their delivery following VR exposure to reduce negative impact and enhance therapeutic effect. Examples of psychological interventions are psychoeducation, emotional and cognitive processing, validation, acceptance and commitment therapy. Similar to the use of VR in exposure therapy for patients with posttraumatic stress disorder, we recommend VR not be in a “self-help” format but with available treatment options for potential therapeutic benefits, which was advocated by Rizzo et al. (30). Fourth, clinical training in psychology may contribute to a therapeutic alliance that helps foster trust and facilitates patients’ willingness to engage fully in VR. Lastly, professional trainings in psychology or related disciplines uphold ethical standards that help safeguard patient privacy, provide informed consent, manage potential risks, and ensure that patients’ psychological well-being and safety are prioritized throughout the VR interventions.

A good attitude from VR therapists is considered important for the quality delivery of VR as well. We advise VR therapists to have reasonable expectations for the efficacy of VR interventions. VR may help patients fulfill their last wishes virtually; however, it is important to note that exposing vulnerable patients to an ideal virtual environment has implications and potential psychological risks. We advocate the importance of clinical sensitivity to the patients’ mental state, psychological readiness for VR intervention, imminent needs, and existential challenges, with a professional demeanor that respects their needs and vulnerability. As patients at their end-of-life stage are particularly vulnerable when facing existential challenges, VR exposure may be an apparent trigger for emotional disturbances. We therefore consider VR therapists’ preparedness and readiness to deliver timely psychological interventions paramount.

### 3.2. Technical role

The technical dimension refers to the technical skills of VR delivery, i.e., the operation of the VR hardware and software. Basic technical skills include operating the VR headset, the remote control, VR apps, products, and content, and an internet connection. As most patients under palliative care are physically fragile, we suggest special precautions to minimize potential physical harm. For instance, we advise VR therapists to locate an appropriate time or private space for VR session to minimize fatigue or exhaustion (i.e., avoiding time right after rehabilitation exercises, medical treatment, or late of the day); to ensure patient in a comfortable lying or sitting position to minimize discomfort due to prolonged posture; to verbally check if patients are feeling uncomfortable on-and-off during VR exposure; to alert patients the possible adverse effects, and prepare them for a “stop” sign if experiencing any discomfort; to minimize risk of cybersickness by limiting VR exposure (31) to about ten minutes, which are observed to be an optimal duration to minimize risk while maintaining therapeutic effect in our palliative care setting; to hold the VR headset for patients in front of their heads to minimize its weight, which was associated with subjective discomfort (32); to screen out patients who may be prone to physical risks, e.g., patients with visual and hearing impairment, epilepsy or having a seizure in the past six weeks, hypersensitivity to motion, active nausea or vomiting, and physical injuries including head wounds, neck pain.

To ensure quality delivery of VR interventions, we suggest VR therapists have basic knowledge of VR technology (i.e., hardware and software), safety measures, ethics concerning data privacy and security, as well as the latest research on VR in palliative care. We consider the following attitudes important: readiness and open-mindedness to keep abreast of updated knowledge and skills of VR in palliative care; adaptability in the face of quick changes in technology; and continual learning, which involves a commitment to ongoing professional development and self-reflection.

### 3.3. Supervisory role

The supervisory dimension refers to the duties of a trainer of VR therapists and VR assistants. As VR has been increasingly practiced in various settings (12, 13, 33, 34), professional trainings for future VR therapists are important to ensure its wide adoption and accessibility. The supervisory role of a VR therapist involves providing guidance, support, and feedback to future VR therapists. The goal of supervision is to help the supervisees develop relevant knowledge, skills, and attitudes for quality VR delivery. We recommend the supervisors to have basic knowledge and skills in VR technology, adequate clinical experiences with VR interventions, and counseling experience in palliative care. Favorable attitudes for a VR therapist include openness, supportiveness, respect, and reflectiveness.

Our clinical experiences show that support from a patient care assistant in operating the VR can improve the efficiency of VR delivery. The trained assistant can perform technical tasks that leave capacity for a VR therapist to focus on clinical tasks, such as clinical observations or interaction with patients. Technical tasks include sanitizing the VR headset for infection control, locating the VR content, and holding the VR headset for the patient to minimize weight during VR exposure. We propose VR therapists to have adequate supervisory skills for both future VR therapists and VR assistants.

## 4. Discussions

Based on the clinical experience of a registered clinical psychologist, we explore the perspective of the required professional training on knowledge, skills, and attitudes for a competent VR therapist. As VR has been increasingly employed for patients with terminal diseases in various settings (12, 13, 33, 34), we highlight the importance of professional training to ensure minimal harm with a positive outcome. Given the scarcity of reports on the required professional training for a competent VR therapist in palliative care, we hope this perspective article opens up discussions and contributes to current understanding and emerging future directions to ensure quality and ethical delivery of VR in palliative care. We also hope to alert future VR therapists early to the potential adverse effects of using VR for patients under palliative care and inspire professional training of VR therapists as an area for further investigation.

## Data Availability

The original contributions presented in the study are included in the article/Supplementary Material, further inquiries can be directed to the corresponding author.
